# Sound frequency affects speech emotion perception: results from congenital amusia

**DOI:** 10.3389/fpsyg.2015.01340

**Published:** 2015-09-08

**Authors:** Sydney L. Lolli, Ari D. Lewenstein, Julian Basurto, Sean Winnik, Psyche Loui

**Affiliations:** Department of Psychology, Program in Neuroscience and Behavior, Wesleyan University, Middletown, CT, USA

**Keywords:** amusia, tone-deafness, pitch, filtering, speech, emotion, frequency

## Abstract

Congenital amusics, or “tone-deaf” individuals, show difficulty in perceiving and producing small pitch differences. While amusia has marked effects on music perception, its impact on speech perception is less clear. Here we test the hypothesis that individual differences in pitch perception affect judgment of emotion in speech, by applying low-pass filters to spoken statements of emotional speech. A norming study was first conducted on Mechanical Turk to ensure that the intended emotions from the Macquarie Battery for Evaluation of Prosody were reliably identifiable by US English speakers. The most reliably identified emotional speech samples were used in Experiment 1, in which subjects performed a psychophysical pitch discrimination task, and an emotion identification task under low-pass and unfiltered speech conditions. Results showed a significant correlation between pitch-discrimination threshold and emotion identification accuracy for low-pass filtered speech, with amusics (defined here as those with a pitch discrimination threshold >16 Hz) performing worse than controls. This relationship with pitch discrimination was not seen in unfiltered speech conditions. Given the dissociation between low-pass filtered and unfiltered speech conditions, we inferred that amusics may be compensating for poorer pitch perception by using speech cues that are filtered out in this manipulation. To assess this potential compensation, Experiment 2 was conducted using high-pass filtered speech samples intended to isolate non-pitch cues. No significant correlation was found between pitch discrimination and emotion identification accuracy for high-pass filtered speech. Results from these experiments suggest an influence of low frequency information in identifying emotional content of speech.

## Introduction

Pitch is a perceptual attribute of sound that allows us to order sounds on a frequency-related scale. It is an integral component of auditory processing, including music and language. Across all spoken languages, pitch is one of several cues used to convey emotional prosody, and in some language (tone languages) pitch is also used to convey meaning in words. Understanding how pitch perception affects our interpretation of speech is essential to fully comprehend the ways in which we communicate emotion through language.

Amusic, or “tone-deaf” individuals, are limited in their ability to perceive and produce pitch (Peretz et al., 2002; Hyde and Peretz, 2004; Vuvan et al., 2015). Though amusia is traditionally thought of as a music-specific disorder, studies have shown that it may also affect perception of speech. In common-practice Western music, pitches typically vary by a minimum of one semitone. In language, intonation patterns that help us discriminate between statements and questions are characterized by pitch differences that range from 5 to 12 semitones, and occur primarily at the conclusion of a speech fragment (Hutchins et al., 2010). By contrast, pitch changes that reflect prosody in emotional speech lie somewhere in between one and five semitones, and occur over the course of a speech fragment, suggesting that pitch variations in emotion expression are harder to detect than question-statement differences (Dowling and Harwood, 1986).

Consistent with this hypothesis, Hutchins et al. (2010) showed that when asked to discriminate between statements and questions, amusics performed as well as controls. However, when asked to judge whether the same stimuli ended with a rising or falling contour, amusics were significantly less accurate and consistent, suggesting a deficit of pitch awareness in amusics. Though amusic subjects self-reported no difficulties during day-to-day speech processing, Jiang et al. (2012) found that amusics’ brain activity was not reliably elicited in response to pitch changes of one semitone in speech [this is in contrast to some early processing of small pitch changes without conscious awareness in music (Peretz et al., 2005, 2009)]. Also, (Nguyen et al., 2009) observed some decreases in sensitivity to pitch inflections found in a tonal language among amusic non-tonal language speakers (Nguyen et al., 2009). Although results from amusics are task-dependent and do overlap with non-amusic controls, studies generally show that amusics have some impairments in speech intonation processing, extending the effects of the disorder beyond music. Other studies have shown that amusics self-report difficulty detecting certain nuances in speech, such as sarcasm, and that they struggle to judge emotional content of speech as accurately as non-amusics (Thompson et al., 2012). In addition, individuals with amusia-like deficiencies report difficulty in determining emotion solely from speech, and may rely more on facial expressions and gestures than control subjects do (Thompson et al., 2012). Though there are other cues in emotional communication that are available to amusics, limitations in the ability to perceive pitch clearly contribute to deficiencies in emotional speech perception.

It has been hypothesized that deficiencies may only be noticeable when amusics are presented with very subtly different stimuli. Liu et al. (2012) presented statement-question discrimination tasks to Mandarin speakers, under conditions of natural speech and gliding tone analogs. Amusics were worse at discriminating gliding tone sequences, and had significantly higher thresholds than controls in detecting pitch changes as well as pitch change directions. However, amusics and controls performed similarly in tasks involving multiple acoustic cues, suggesting that instead of using fine-grained pitch differences to interpret meaning, individuals with pitch perception deficits might have relied on some non-pitch cues. In another study, Liu et al. (2010) presented similar statement-question discrimination tasks under the conditions of natural speech, gliding tones, and non-sense speech analogs. Amusics performed significantly worse than non-amusic control participants in discrimination under all three conditions, suggesting deficiencies not only in samples with isolated pitch contour, but also in natural speech. Liu et al. (2015) again examined this link between amusia and speech processing in Mandarin speakers using speech samples with normal or flattened fundamental frequency contours. Amusics showed reduced speech comprehension when listening to flattened samples in quiet and noisy conditions, while controls only showed reduced speech comprehension in noisy conditions, suggesting that amusics experience speech comprehension difficulties in everyday listening conditions, with deficits extending to impaired segmental processing, rather than being limited to pitch processing.

Our study aims to analyze the extent of impairment in more nuanced areas of speech, namely emotional recognition. It has been suggested that individuals may compensate for poor pitch perception by relying more heavily on alternative cues within speech to infer emotional content, such as stress and emphasis (Hutchins et al., 2010). Speech segments that express five emotions (happy, sad, irritated, fearful, tender) and no emotion are presented as both filtered and non-filtered stimuli to participants. Rather than focusing exclusively on amusic populations, our goal is to test how individual differences in pitch perception can impact the processing of emotional prosody.

Frequency filtering methods are often used in tests that diagnose deficits in auditory perception, in order to simulate subtle differences in music and speech content (Patel et al., 1998; Ayotte et al., 2002; Bhargava and Başkent, 2012; O’Beirne et al., 2012). Low-pass filters may be used to examine speech intelligibility independently or in conjunction with other auditory disturbances (Horwitz et al., 2002; Bhargava and Başkent, 2012). The majority of speech prosody cues are preserved, while speech intelligibility is lost, with a sharply sloped low-pass filter around 500 Hz (Knoll et al., 2009; Guellaï et al., 2014). In our first experiment we applied a low-pass filter that attenuates frequencies above 500 Hz to disrupt intelligibility while still maintaining the fundamental frequency of speech sounds, which gives rise to their pitch contour. In our second experiment, we applied a high-pass filter in order to retain cues other than pitch contour, such as accents and sibilants, which may provide emotional cues. High-pass filters have been used in previous studies, but rarely in amusic populations. Our filter attenuated frequencies below 4800 Hz, providing the listener with minimal pitch contour while preserving rhythmic structure and sibilants.

Natural speech contains many cues that amusics can perceive, prompting them to report predominantly normal speech perception. Studies suggest that amusics who do not report deficiencies in everyday speech may more heavily weigh tempo, mode, and linguistic content in processing emotional significance (Peretz et al., 1998; Gosselin et al., 2015). Low-pass and high-pass filtered speech, in contrast to natural, unfiltered speech, contain less information to factor into individuals’ interpretation of emotional content. We hypothesize that there will be a negative correlation between pitch discrimination thresholds and accuracy in emotional identification under low-pass conditions, i.e., that individuals with poorer pitch perception skills are less able to use low-frequency speech cues to identify emotional prosody. We also hypothesize that unlike low-pass filtering, high-pass filtering speech samples will not affect emotional identification disproportionately for poor pitch perceivers.

## Norming Study

The Macquarie Battery for Evaluation of Prosody (MBEP) has been used in previous experiments to assess the effects of amusia on emotional prosody perception (Thompson et al., 2012). The Macquarie database was created from semantically neutral statements (e.g., “The broom is in the closet and the pen is in the drawer”), read by four male and four female actors to represent no emotion and five different emotions (happy, sad, tender, irritated, and frightened). The statements are 14 syllables long, and the emotions were chosen for the variety of acoustic cues that they offer. In total, the database included 96 recorded statements. The statements in the MBEP were recorded in Australia, and thus are recorded with an Australian accent. We performed a norming study on Amazon Mechanical Turk to ensure that American subjects would be able to properly identify emotion in Australian-accented speech.

### Methods

Ninety-six statements from MBEP were presented as separate, single-question surveys on Amazon’s Mechanical Turk, and subjects were allowed 1 min to listen and respond by identifying the emotion. Subjects were paid $0.05 per question. Each of the 96 statements in the database received 10 responses from users in the United States.

### Results

**FIGURE 1:**
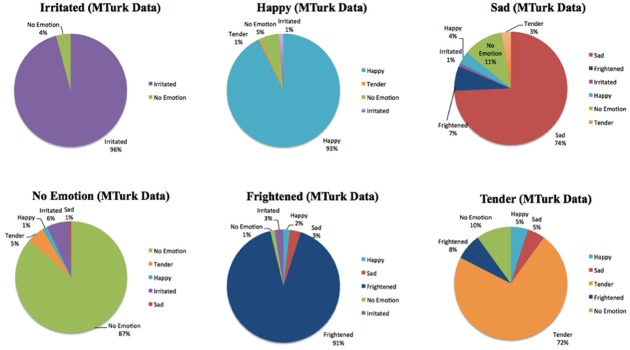
**Emotional identification results from Mechanical Turk listeners of MBEP speech samples**.

Results from the norming study are shown in Figure 1. Subjects performed well above chance levels in all emotional categories, confirming that American subjects were able to identify emotion in Australian-accented speech.

### Discussion

Listeners were reliably successful at identifying the intended emotion from MBEP speech samples. “Irritated” was the most commonly correctly identified emotion, while “tender” was the least commonly correctly identified emotion. Based on listeners’ responses, statements in which respondents chose the target emotion less than 50% (chance level = 16.7%) of the time were excluded from use in the study. Tender statements were more likely than other emotions to be excluded, as they were typically more difficult to identify. Twelve statements from the set were excluded from use, resulting in 84 speech samples in the rest of the study. These 84 speech samples included 16 of Happy, Frightened, Irritated, and No Emotion, 14 Sad samples, and six Tender samples.

## Experiment 1: Low-Pass Filter

### Materials and Methods

#### Participants

Forty participants (21 women and 19 men) aged 18–22 from an introductory psychology course at Wesleyan University participated in exchange for course credit. All participants gave informed consent as approved by the Psychology Ethics Board of Wesleyan University. Participants reported no hearing impairment, neurological disorders, or psychiatric disorders. Twenty-five of the forty participants reported musical training with varying instruments for lengths of time ranging from 6 months to 13 years. Across participants with previous musical training, an average of 6.5 years of training was reported. All subjects took the Montreal Battery of Evaluation of Amusia (MBEA) as well as the pitch discrimination test. Pitch discrimination thresholds, as identified by the pitch discrimination task (described below), ranged from 1.5 to 48 Hz (mean = 10.5 Hz). Nine subjects were considered amusic based on their inability to identify differences in pitch greater than 16 Hz apart (at 500 Hz) in the pitch discrimination task (amusic mean = 23.2 Hz, SD = 10.4 Hz; control mean = 6.8 Hz, SD = 3.9 Hz). Fifteen subjects were considered amusic based on their scores on the MBEA contour subtest (fewer than 23 correct responses out of 31 possible). Four subjects failed both the pitch discrimination threshold test and the MBEA. While the MBEA and pitch discrimination test both measure aspects of musical perception, especially pitch perception, MBEA is broader and also measures attention and working memory. Here we rely on the pitch discrimination test because we are interested more specifically in pitch discrimination aspects of musical function, rather than the attention and working memory components.

#### Materials

Several tests were administered to assess musical ability and training: the contour subtest of the a pitch discrimination threshold test MBEA, a questionnaire on demographic information and musical training, and the Shipley Institute of Living Scale (Shipley, 1940), used as a non-verbal IQ control task as it has been shown to be a predictor of WAIS-IQ scores (Paulson and Lin, 1970). Amusia was measured using the contour subtest of the MBEA (Peretz et al., 2003) and a pitch discrimination task. In the contour subtest, two brief melodies are presented that are either identical or differ to varying degrees in pitch contour. The pitch discrimination threshold test (Loui et al., 2008) determines the smallest pitch interval that participants are able to distinguish by presenting a series of two tones and asking whether the second tone is higher or lower in pitch than the first. The test uses a three-up one-down staircase procedure to find the threshold range of pitch perception. The questionnaire administered to the participants included questions about the following: sex, date of birth, first languages, and history of hearing impairment, neurological disorders, or psychological disorders. The questionnaire also included questions on participants’ musical training history. If the subject responded that they had trained on an instrument, he or she was asked to share the length of training, age of onset, and the instrument(s) trained on.

**FIGURE 2:**
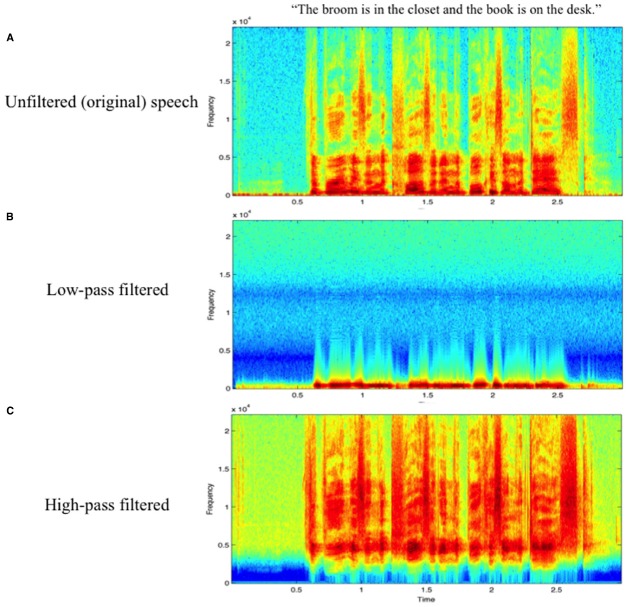
**Spectrograms of a representative speech sample in (A) unfiltered, (B) low-pass filtered, and (C) high-pass filtered conditions**.

A behavioral test was then administered using 84 non-filtered and 84 low-pass filtered speech samples from the MBEP, chosen from the norming study reported above. The non-filtered trial condition consisted of natural (unfiltered) speech samples directly from the database, excepting 12 samples that Mechanical Turk workers did not reliably identify with above 50% accuracy. The low-pass filtered trial condition consisted of frequency-filtered versions of the same 84 speech samples, filtering out frequencies above 500 Hz. Filtering was done in Logic X with the plugin “Channel EQ” (Q factor = 0.75, slope = 48 dB/Octave). This low-pass filtered condition was intended to eliminate formants and other high-frequency cues from the speech samples, while preserving the pitch contour of the speech samples. See Figure 2 for spectrogram representations of unfiltered (Figure 2A) and low-pass filtered (Figure 2B) speech samples.

#### Procedure

Participants were individually administered the tests as stated above in a laboratory setting with minimal noise interference. Stimuli were presented through Sennheiser 280 HD Pro headphones connected to a desktop iMac computer at a comfortable volume for the subject. The experiment was created using Max/MSP and the two trial blocks were presented in a randomized order, with the aim of balancing out any potential order effects of the blocks. All subjects were equally likely to start on unfiltered and filtered speech. The speech samples within each trial block were also presented in a randomized order. Subjects used the mouse to choose one of the six emotion categories listed from among six options: Happy, Sad, Irritated, Frightened, Tender, and No emotion.

#### Data Analysis

Data were exported from the experiment in Max/MSP to Excel and SPSS for analysis. Pitch discrimination thresholds were log-transformed (log base 10) to achieve normal distribution.

## Results

**FIGURE 3:**
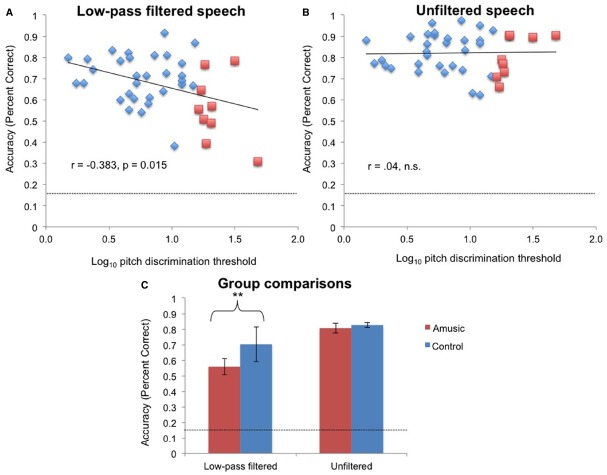
**The relationship between log pitch discrimination threshold and emotional identification accuracy (A) in the low-pass condition and (B) in the unfiltered speech condition.** Red squares: amusics; blue diamonds: controls. Dashed line indicates chance performance. **(C)** Accuracy in emotional identification in amusics and control subjects. **p < 0.01.

Log pitch discrimination threshold was significantly correlated with emotional identification accuracy in the low-pass filtered condition [*r*(38) = –0.38, *p* = 0.015; Figure 3A] but not in the unfiltered speech condition [*r*(38) = 0.04, n.s.; Figure 3B].

Amusics (as identified by pitch discrimination thresholds) performed worse than controls in the filtered condition [*t*(38) = –3.13, *p* = 0.003], but not in the unfiltered speech condition [*t*(38) = –0.58, n.s.; Figure 3C]. When amusics were identified using the contour subtest of the MBEA, their performance in the low-pass filtered condition was still below that of controls (amusics mean = 62%, SD = 16%; controls mean = 70%, SD = 12%); however the difference was only marginally significant [*t*(38) = 1.7, *p* = 0.09]. Amusics identified using the MBEA contour test did not differ in performance from controls in the unfiltered speech condition [amusics mean = 84%, SD = 10%, controls mean = 81%, SD = 9%, *t*(38) = 1.14, *p* = 0.26].

When holding musical training constant in a partial correlation, accuracy under low-pass conditions was still correlated with pitch discrimination threshold [*r*(37) = –0.35, *p* = 0.028] and unfiltered speech condition accuracy remained uncorrelated [*r*(37) = –0.04, n.s.]. These results confirm that even when controlling for musical training, pitch perception was significantly correlated with emotional identification accuracy under low-pass filtered but not under unfiltered speech conditions. When controlling for Shipley Abstraction scores, the correlations hold at *r*(37) = –0.38, *p* = 0.018 for the low-pass condition, and *r*(37) = –0.04, n.s. for the unfiltered speech condition. Using both Shipley scores and musical training as control variables, accuracy in the filtered condition remained correlated with pitch discrimination scores [*r*(36) = –0.35, *p* = 0.033] and unfiltered speech accuracy remained uncorrelated [*r*(36) = 0.03, n.s.].

As subjects were randomly assigned to begin the experiment with the low-pass filtered block (*n* = 19, 6 amusics) or the unfiltered block (*n* = 21, 3 amusics), it was possible for block order to have influenced results: specifically, experience with the unfiltered speech condition could have helped a subject’s subsequent performance on the low-pass condition. A follow-up analysis was conducted to assess the effects of block order on performance in the low-pass filtered condition. Order was incorporated as a variable in a between-subject ANOVA. A two-way ANOVA on the dependent variable of accuracy in the low-pass condition, with the factors of group (amusics vs. controls) and block order (low-pass first vs. unfiltered speech first) showed a significant main effect of amusia [*F*(1,36) = 5.5, *p* = 0.025] and a significant main effect of block order [*F*(1,36) = 7.3, *p* = 0.01], as well as a significant interaction between amusia and block order [*F*(1,36) = 4.8, *p* = 0.034]. In addition to confirming that amusics performed worse at emotional identification in low-pass filtered speech, this result suggests that subjects learned to identify emotions via prosody throughout the course of the experiment: those who started with unfiltered speech subsequently performed better on the low-pass filtered condition, compared to those who started on the low-pass filtered condition, presumably because subjects learned during the unfiltered speech condition to listen for pitch as an emotional cue. Interestingly, the significant interaction between group and block order shows that the amusics who started on the low-pass condition performed worse than the amusics who started on the natural speech condition, who were indistinguishable in performance from controls. This interaction suggests that learning throughout the experiment may occur even more in amusics than in controls.

Scores on the MBEA showed no significant correlation with emotional identification accuracy in the low-pass filtered condition [*r*(38) = 0.18, n.s.]. MBEA was not correlated with emotional identification accuracy under unfiltered speech conditions [*r*(38) = –0.04, n.s.]. Amusics (as identified by MBEA score) did not perform significantly differently between the filtered condition [*t*(37) = –0.33, n.s.] and the unfiltered speech condition [*t*(38) = 1.20, n.s.].

## Discussion

Results show a robust association between pitch perception ability and accuracy of emotional identification in speech in the low-pass filtered conditions, but not in unfiltered speech. Amusic individuals, identified as those who have poor pitch perception abilities, are impaired in identifying the emotional content of speech when high-frequency cues are removed from the speech. These individual differences are uniquely related to pitch discrimination abilities, and are not explained by differences in general IQ or musical training.

Given the dissociation between low-pass filtered and unfiltered speech conditions, we inferred that amusics may be compensating for poorer pitch perception by using speech cues that are filtered out in the former manipulation. To assess this potential compensation, a second experiment was conducted, using high-pass filtered speech samples intended to isolate non-pitch cues.

## Experiment 2: High-Pass Filter

### Materials and Methods

#### Participants

Twenty-nine participants (17 women and 12 men) aged 18–28 from an introductory psychology course at Wesleyan University participated in exchange for course credit. Participants reported no hearing impairment, neurological disorders, or psychiatric disorders. Twenty-one of the 27 participants reported musical training with varying instruments for lengths of time ranging from 1 to 11 years. Among participants with previous musical training, an average of 5.6 years of training was reported. All subjects took the Montreal Battery as well as the pitch discrimination test. Pitch discrimination thresholds, as identified by the pitch discrimination task (described below), ranged from 1.3 to 27.5 Hz (mean = 10.5 Hz). Three participants were considered amusic based on their inability to identify differences in pitch greater than 16 Hz apart (at 500 Hz) in the pitch discrimination task (amusic mean = 26 Hz, SD = 2.1 Hz; control mean = 7.8 Hz, SD = 4.3 Hz). Twelve participants were considered amusic based on their scores on the MBEA contour subtest (fewer than 23 correct responses out of 31 possible). Three participants failed both the pitch discrimination and the MBEA tests.

#### Materials

The tests used to assess musical ability and training and the Shipley Institute of Living Scale were the same as administered in Experiment 1. A behavioral test of emotional identification was then administered using the same 84 unfiltered (original) speech samples from the MBEP (the same unfiltered speech samples used in Experiment 1, chosen from the norming study reported above), and 84 new high-pass filtered speech samples generated for this experiment. Filtering was done in Logic X with the plugin “Channel EQ” (Q factor = 0.75, slope = 48 dB/Octave). The frequency cutoff for high-pass filtering was chosen at 4800 Hz (i.e., frequencies lower than 4800 Hz were attenuated) to eliminate cues such as pitch contour and the majority of formant frequencies, while preserving other cues such as speech rate, stress patterns, and rhythm.

#### Procedure

Stimuli were presented through Sennheiser 280 HD Pro headphones connected to a desktop iMac computer at a comfortable volume for the subject. The main experiment was created using Max/MSP and the two trial blocks were presented in a randomized order to the participant. The speech samples within each trial block were also presented in a randomized order. Subjects used the mouse to choose one of the six emotion categories as in Experiment 1.

#### Data Analysis

As in Experiment 1, data were exported from the experiment in Max/MSP to Excel and SPSS for analysis. Pitch discrimination thresholds were log-transformed (log base 10) to achieve normal distribution.

## Results

**FIGURE 4:**
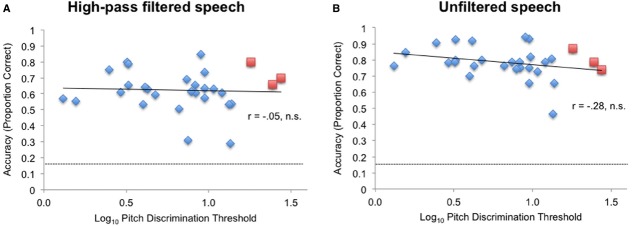
**The relationship between log pitch discrimination threshold and emotional identification accuracy (A) in the high-pass condition and (B) in the unfiltered speech condition.** Red squares: amusics; blue diamonds: controls. Dashed line indicates chance performance.

As shown in Figures 4A,B, pitch discrimination threshold was not significantly correlated with accuracy under high-pass conditions [*r*(27) = –0.05, n.s.], or with accuracy under unfiltered speech conditions [*r*(27) = –0.28, n.s.]. MBEA was also not significantly correlated with overall accuracy of subjects under unfiltered speech conditions or under high-pass conditions.

While it appears that the high-pass filtering manipulation on the speech samples did not result in the same sensitivity to pitch discrimination differences compared to the low-pass filtered speech in Experiment 1, an additional possibility was that differences between the two experiments resulted from using different subjects between the two experiments, i.e., a sampling difference, which is potentially a confound especially since there were only three subjects who met the pitch-discrimination threshold criterion for amusia within the sample of Experiment 2. In a follow-up analysis to test the equivalence of samples between Experiments 1 and 2, we chose a subset of subjects from among our subjects in Experiment 1 who were matched for pitch discrimination thresholds, Shipley scores, and musical training to our subjects in Experiment 2, thereby repeating our analysis with only 3 amusics. A significant negative correlation was still observed between log pitch discrimination threshold and accuracy in the low-pass filtered speech condition, even within this reduced subset of the Experiment 1 sample [*r*(27) = –0.37, *t*(27) = 2.07, *p* = 0.048]. This confirms that the samples of amusic and control subjects are comparable between the two experiments, and that the difference in data pattern between Experiments 1 and 2 is due to our experimental manipulations of the speech samples rather than to sampling differences between the experiments.

## Discussion

Results showed no significant relationship between emotional identification accuracy and individual differences of pitch discrimination, in either the unfiltered speech or the high-pass filtered speech conditions. Although only three of the 29 subjects in this experiment showed pitch discrimination thresholds that exceeded the cutoff for amusia, a continuum of individual differences in pitch discrimination was captured in the present sample. High-pass filtering the speech samples did not result in any positive relationship between emotional identification and pitch discrimination, suggesting that individuals with poor pitch perception were not systematically using high-frequency information in speech as a potential source of compensatory cues toward emotional identification. Importantly, results were not explained by sampling differences between Experiments 2 and 1, as a matched subset of data from Experiment 1 replicated the negative correlation in the low-pass filtered condition that was not observed in the high-pass filtered condition in Experiment 2.

## General Discussion

Results showed a significant negative correlation between pitch discrimination thresholds and emotional identification for low-pass filtered speech, but not high-pass filtered or unfiltered speech. Subjects with poor pitch perception, especially amusics, performed worse than their counterparts in identifying emotions from speech, but only when the speech was low-pass filtered. Amusics were defined here as those with a pitch discrimination threshold of >16 Hz, resulting in nine identified amusics in Experiment 1 and three subjects identified as amusics in Experiment 2. The behavioral dissociation between low-pass and unfiltered speech conditions suggests that low frequency energy bands in speech carry important emotional content, to which amusics are less sensitive.

In the low-pass filtered condition, the observed correlation between emotional identification accuracy and individual differences in pitch discrimination threshold was significant even after controlling for IQ and musical training. This finding suggests that individual differences in pitch perception can exist above and beyond differences in cognitive capacity and musical training, and can have far-reaching consequences that generalize to domains of life beyond musical ability. However, unlike previous reports (Thompson et al., 2012), we did not observe a significant relationship between emotional identification accuracy and pitch discrimination threshold in unfiltered speech. While further work is needed to explain the differences in experiment design that might give rise to our different findings, the observed dissociation from the current study between low-pass filtered and unfiltered speech conditions supports the hypothesis that amusics could have been compensating for their poorer pitch perception in low frequency sounds by using other cues in the speech stimuli. However, the high-pass filtering manipulation (Experiment 2) did not reveal more reliance on high frequency speech cues among poorer pitch perceivers. This may suggest that frequencies above 4800 Hz (the chosen cutoff for high-pass filtering in Experiment 2) were also not the primary source of the compensatory information in speech that amusics might be using to approach the task of emotional identification. Alternately, both groups were using other cues in speech, not captured in the filters used in these studies, to accomplish the task of emotional identification.

Pitch discrimination thresholds were used to define amusia in these experiments rather than the MBEA, as the latter focuses more on melodic discrimination than on individual differences in pitch discrimination *per se*. While amusic participants performed worse in low-pass trials, accuracy for all participants was well above the chance level of 16%. This finding implies that while the fundamental frequency (below 500 Hz) provides some prosodic information such as pitch contour, cues that exist in the range of frequencies between 500 and 4800 Hz may provide further prosodic cues. These midrange frequencies may have been used for emotion recognition in music, in light of recent findings that amusics are able to show normal recognition of musical emotions (Gosselin et al., 2015). Results are also consistent with recent reports showing that amusia is limited to resolved harmonics (Cousineau et al., 2015). Given these results, examining specific frequency bands for prosodic cues may reveal more in the future about the cues that amusics could be using to identify emotions, and to understand speech and music in communication more generally.

Insight into several additional questions may lead to a more complete model explaining this relationship between pitch discrimination and emotional identification. It remains to be determined if there is a causal link between poor pitch perception and poor emotional recognition, or if a third underlying process leads to both deficiencies, as posited by the musical protolanguage hypothesis (Thompson et al., 2012). Poor pitch perception is associated with multiple behavioral and neural differences, such as differences in neural connectivity (Loui et al., 2009), pitch awareness (Loui et al., 2008; Peretz et al., 2009), learning ability (Loui and Schlaug, 2012), and working memory (Williamson and Stewart, 2010), and different contributions of one factor or another may further affect prosodic recognition.

In that regard, one factor that may affect prosodic recognition is learning differences, which was addressed in a follow-up analysis looking at order effects. This showed a significant interaction between amusia and block order: amusics who started the experiment by listening to low-pass filtered speech performed worse than other amusics who started on unfiltered speech. This interaction suggests that learning throughout the experiment may occur even more in amusics than in controls. While more studies are needed to address this possibility in the future, learning could potentially be one of the compensatory mechanisms that amusics use to approach the task of emotional identification when pitch perception is impaired.

Given that a significant correlation between pitch discrimination ability and emotional recognition accuracy was found only when high frequency bands were removed, the data suggest that higher frequency information must have played a role in accurate recognition. Further studies may benefit from examining whether these trends are present among all amusics, or whether in-group distinctions can be made between different amusic individuals. Amusia may be a complex class of disorders with subtle disabilities that are currently categorized under a single category. Related symptoms of amusia, such as rhythmic disabilities, poor singing ability, and deficiencies in musical memory, may be examined to determine if these types of disabilities also correlate with deficiencies in recognition of emotional prosody. By investigating emotional identification in speech by individuals with various musical difficulties, future results may contribute further to the debate on the origins of music and language.

## Conclusion

The present study investigated the relationship between pitch perception and emotional identification in speech. Using a battery of speech that was spoken with different emotional prosody, we showed that poor pitch perception is correlated with lower accuracy in emotional identification tasks, but only for low-pass filtered speech, and not for high-pass filtered or unfiltered speech. The relationship between pitch discrimination and emotional identification accuracy is not explained by differences in IQ and musical training. Future research should be focused toward identifying which speech cues are used by amusics in order to compensate for impaired pitch perception.

### Conflict of Interest Statement

The authors declare that the research was conducted in the absence of any commercial or financial relationships that could be construed as a potential conflict of interest.
